# Insomnia Prevalence among Italian Night-Shift Nurses

**DOI:** 10.3390/nursrep11030050

**Published:** 2021-07-12

**Authors:** Nicola Ielapi, Michele Andreucci, Umberto Marcello Bracale, Davide Costa, Egidio Bevacqua, Andrea Bitonti, Sabrina Mellace, Gianluca Buffone, Stefano Candido, Michele Provenzano, Raffaele Serra

**Affiliations:** 1 Department of Public Health and Infectious Disease “Sapienza” University of Rome, 00185 Rome, Italy; 2 Interuniversity Center of Phlebolymphology (CIFL), International Research and Educational Program in Clinical and Experimental Biotechnology, Headquarters: University Magna Graecia of Catanzaro, Viale Europa, 88100 Catanzaro, Italy; davidcosta3@libero.it (D.C.); egidiobevacqua@unicz.it (E.B.); 3 Department of Health Sciences, University of Catanzaro, 88100 Catanzaro, Italy; andreucci@unicz.it; 4 Department of Public Health, University of Naples “Federico II”, 80100 Naples, Italy; umbertomarcello.bracale@unina.it; 5 Department of Law, Economics and Sociology, University Magna Graecia of Catanzaro, 88100 Catanzaro, Italy; 6 Department of Medical and Surgical Sciences, University of Catanzaro, 88100 Catanzaro, Italy; michiprov@hotmail.it; 7 Private Office, 80100 Rome, Italy; andrea.bitonti@uniroma1.it; 8 Department of Patient’s Service, Civic Health Agency of Trento, 38122 Trento, Italy; mellace.sabrina@gmail.com; 9 Department of Vascular Surgery, Health Agency of Trento, 38122 Trento, Italy; gianlucabuffone@gmail.com; 10 Intensive Care Unit, Pugliese Ciaccio Hospital of Catanzaro, 88100 Catanzaro, Italy; candiste@libero.it

**Keywords:** insomnia, night shift, nursing, prevalence, Italy

## Abstract

Background. Insomnia is one of the major health problems related with a decrease in quality of life (QOL) and also in poor functioning in night-shift nurses, that also may negatively affect patients’ care. The aim of this study is to evaluate the prevalence of insomnia in night shift nurses. This observational online web-based survey using Google^®®^ modules specifically aimed to investigate the prevalence and risk factors for insomnia among Italian nurses. Methods. Data collection for this study lasted one month, with the questionnaire available from 1 March 2021 to 1 April 2021. Continuous variables were considered as either mean ± standard deviation (SD) or median and interquartile range (IQR) based on their distribution. Comparison among insomnia categories was assessed by one-way ANOVA or Kruskal–Wallis test according to variable distribution. Categorical variables were analyzed using chi-square test. Results. A total of 2355 responses were included in the final analysis, with 917 from the Northern zone, 815 from the Western zone, and 623 from the Southern zone of Italy. The prevalence of insomnia in the overall population was 65.4% (1524 out 2355 nurses suffered from insomnia). Conclusions. Nursing is a high-pressure profession, with heavy duties and high professional risks. We found an important prevalence of insomnia in night shift nurses, and we hope it may help to solicit further studies aimed to identify the risk factors for this working disorder among nurses.

## 1. Introduction

Sleep deprivation as a result of working during the night may predispose workers to insomnia [1], a sleep disorder that afflicts between 6% and 10% of the general adult population in industrialized countries [2]. Insomnia is one of the main causes of depressive disorders and is related with a decrease in quality of life (QOL) as well as with poor functioning in the workplace [3]. Work hours that result in a non-standard sleep–wake cycle, due to shorter sleep duration and/or sleepiness, can cause serious sleep alterations affecting individuals’ sleep circadian system [4]. Working in the health sector is often associated with night shifts, especially for nurses who have a significant role in direct patient care [5]. Nurse fatigue after night shifts might be associated not only with significant risks to patient safety and their management, but also to their own safety in the case of situations such as driving home post-shift [6].

## 2. Study

### 2.1. Aim

The aim of this study is to evaluate the prevalence of insomnia in night shift nurses.

### 2.2. Study Design and Procedures

We performed an observational online web-based survey using Google^®®^ Modules specifically aimed to investigate the prevalence and risk factors for insomnia among nurses working in Italian hospitals. To overcome the temporal bias of enrolment, data collection for this study lasted one month, with the questionnaire available from 1 March 2021 to 1 April 2021. Given the historical moment of collection, several questions have also been oriented toward the comprehension of the influence of the COVID-19 pandemic on the burden of insomnia in this specific population. All nurses aged 25–65 years, who were active working in Italian hospitals at the time the questionnaire was sent have been considered eligible for the study and thus included in the survey. With the aim of standardizing the sample, online platforms were searched by name of different Italian zones. Next, zones were used to stratify the sample. The participants were contacted randomly through their available contact information, namely e-mail, Facebook or WhatsApp number, and the Google form was shared. Insomnia was detected via the individual perception of difficulty falling asleep and/or the sleeping pill consumption.

This work fully complies with STROBE guidelines (https://www.strobe-statement.org/index.php?id=strobe-home accessed on 5 July 2021) [7].

### 2.3. Ethical Considerations

The study was approved by the Institutional Review Board (IRB) of CIFL (approval number: E.R.ALL.2018.43.A), and all the nurses who participated in the survey gave online informed consent.

### 2.4. Statistical Analysis

Continuous variables were reported as either mean ± standard deviation (SD) or median and interquartile range (IQR) based on their distribution. Comparison among insomnia categories was assessed by one-way ANOVA or Kruskal–Wallis test according to variable distribution. Categorical variables were analyzed using chi-square test. For the model building process, univariate analysis testing the association between the main variables and insomnia, modeled as a categorical variable, was assessed by means of logistic regression analysis. The variables with *p* < 0.15 at univariate analysis were selected and included in the first multivariate logistic regression model (Model 1). Next, backward variable selection method with an elimination criterion of *p* ≤ 0.05 was performed to fit the second multivariate logistic regression model (Model 2). Multicollinearity was assessed with variance inflation factors (VIF), which are a measure of the degree to which a single predictor variable can be expressed as a linear combination of the remaining predictor variables; values greater than 10 were cause for concern [8]. The final model (Model 2) was adjusted by: age, smoking habit, afternoon resting, history of insomnia previous to start working, referral to a medical specialist, impairment (or new onset) of insomnia symptoms attributed to COVID-19 period, changing bedtimes during COVID-19 period. Data were analyzed using STATA version 16 (Stata Corp., College Station, TX, USA).

## 3. Results

A total of 2355 responses were included in the final analysis, with 917 from the Northern zone, 815 from the Western zone, and 623 from the Southern zone of Italy.

**Figure 1:**
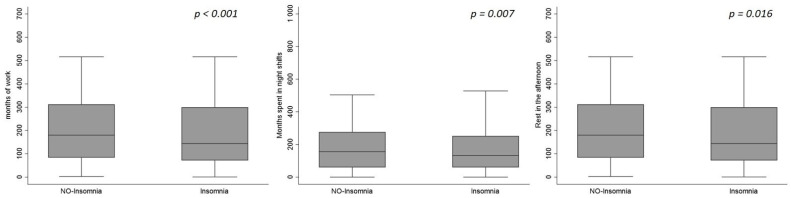
Box plots representing the associations between variables related to work and the presence of insomnia.

The overall cohort was characterized by a relatively young age (40.4 years on average), and a non-trivial frequency of current smokers (34.6%). The cumulative worktime for all participants was 168 [72–300] months, Table 1. The prevalence of insomnia in the overall population was 65.4% (1524 out 2355 nurses suffered from insomnia). When we compared participants’ characteristics among insomnia and NO-insomnia categories, we found that participants with insomnia were significantly younger than those without (39.9 ± 10.5 vs. 41.4 ± 10.0, *p* = 0.001), were more frequently smokers (37.2% vs. 29.5%, *p* < 0.001) and had also a significantly shorter worktime (144 vs. 180 months on median, *p* < 0.001). Moreover, participants with insomnia were characterized by a shorter duration of time spent in night shifts (*p* = 0.007) and by a lower period of rest in the afternoon (*p* = 0.016, Table 1 and Figure 1). A larger prevalence of participants with insomnia reported an impairment or even new onset of symptoms during COVID-19 pandemic (82.5% of participants with insomnia vs. 61.4% without insomnia, *p* < 0.001) and most of them attributed this phenomenon to the pandemic itself. When the variables with a weak-moderate association (*p* < 0.150) with insomnia have been included in the multivariable model (Model 1), smokers, nurses with a history of insomnia previous to start working, and those who needed to refer to a medical specialist were significantly associated with the presence of insomnia (Table 2). In the same model, the association between insomnia and the impairment (or new onset) of insomnia symptoms during the COVID-19 period, the attribution of such impairment to pandemic and the changing of bedtimes during the COVID-19 period was highly statistically significant (*p* < 0.001). After the stepwise selection, which retained in the model the more significantly associated variables with the presence of insomnia (Model 2), we found that young age (*p* = 0.014), smoking habit (*p* = 0.005), short afternoon resting (*p* = 0.05), history of insomnia previous to start working (*p* < 0.001), and referral to a medical specialist (*p* = 0.017), were independent strong correlates of the presence of insomnia. Still in Model 2, impairment (or new onset) of insomnia symptoms during the COVID-19 period, attribution of these symptoms’ impairment to the COVID-19 pandemic and changing bedtimes during the COVID-19 period were strong risk factors for the presence of insomnia (*p* < 0.001 for all variables). When VIF was computed for both models, it was considered acceptable, being lower than five for all variables. Thus, we excluded collinearity with great confidence.

## 4. Discussion

In Western countries, generally only 27% of employed people work the classic normal daytime employment time, and more than 20% work on shifts, including night work [1,4].

Health care personnel represent the largest global workforce that are more likely to be night shift workers and approximately half of all health care workers are nurses [9,10].

Night shift working is a well-known risk factor for disturbed circadian rhythm leading to sleep disorders, with shorter sleep duration, sleepiness, and a generally affected ability to sleep determining insomnia [1,3,4].

After a night shift, nurses are likely to sleep during the daylight phase of biological rhythms, which is characterized by wakefulness. Moreover, lighting and noise during the daylight phase make falling asleep even more difficult and unfavorable. As a result, the duration of sleep is decreased by 2–4 h or even prematurely interrupted, with important alterations of nonrapid-eye-movement (NREM) and rapid-eye-movement (REM) sleep phases. These conditions in the long term may lead to persistent and severe sleep disturbances with chronic fatigue, anxiety and depression, and also may cause fluctuations of alertness and vigilance resulting in work accidents, traffic accidents, and most of all, in the case of health personnel, significant risks to patients’ safety and their management [1,6].

Disturbed circadian rhythm due to night shift affects workers’ daily life functioning and may also predispose them to several chronic diseases such as type 2 diabetes mellitus, obesity, hypertension, gastrointestinal tract disorders, cardiovascular disease, mood disorders, anxiety, depression and women’s hormonal and reproductive function [1,3].

In our study, we found an important prevalence of insomnia (65.4%) in relatively young workers (40.4 years on average). This estimate is higher than that observed in other clinical or demographic settings [11,12]. In a study enrolling about 60 thousand subjects selected from the general population in 13 countries around the world, prevalence of insomnia was 11.3% [11]. A variable frequency of diagnosed insomnia, ranging from 5% to 48%, has been reported elsewhere [13,14,15]. However, our findings are hardly comparable with the general population. In fact, general population surveys include heterogeneous subjects with different risk factors at baseline. For example, in the international survey of Aernout et al., more than 11,000 patients were classified as “retired from work” and, curiously, this category showed a statistically significant protection from insomnia [11]. In our study, all the nurses enrolled were active workers and this can, at least in part, explain the higher frequency of insomnia.

Another non-trivial factor that could have impacted on the estimate of insomnia is the influence of the COVID-19 pandemic. In fact, it has been already shown in a recent meta-analysis that healthcare workers were more vulnerable to stress condition and sleep disturbance during the pandemic [16]. Most importantly, this trend referred to both nurses and physicians. We also detected a significant and independent association between the COVID-19 pandemic and worsening or new onset of insomnia in our cohort. The population of nurses has been considered as a high-risk setting for the onset of insomnia. Among Polish nurses involved in a recent survey (2017), half of the workers suffered from insomnia [17]. Moreover, in this study the presence of insomnia was not related to the night shift rotation. This fits with our results. In this context, we even found an inverse association between age and the presence of insomnia at multi-adjusted analysis, thus meaning that prevalence of insomnia decreases as age increases. We may hypothesize that workers with less experience are more overburdened by work and related anxiety, which may in turn represent a trigger for insomnia.

In our data, nurses suffering from insomnia were more frequently smokers with also a significantly shorter worktime. Such association between smoking and insomnia is relevant when considering that smoking habit increases per se the cardiovascular and mortality risk [18]. Even more importantly, this should be considered in combination with the finding that insomnia is an independent risk factor for health impairment [19].

## 5. Conclusions

In our study, we found an important prevalence of insomnia in night shift nurses.

This may also probably be due to their high occupational stress compared to other health professionals. In fact, nursing is a high-pressure profession, with heavy duties, high professional risks and with low salaries compared to those of physicians, and all these factors may lead to the increase of working pressure with consequent sleep disorder onset [20].

Further studies are needed in order to follow up, over time, this relationship, and to identify the risk factors that make nurses more sensitive to this disorder, and consequently to implement management strategies to improve quality of life of night shift nurses, such as improving physical health, improving nutrition and fitness, and also providing better night work settings [21].

## Figures and Tables

**Table 1:** Clinical and demographic characteristics of subjects enrolled in the study. The features are presented for the whole study population (on the left) and according to the presence or absence of insomnia.

Questions	Overall (*n* = 2′355)	Insomnia (*n* = 1′542)	NO-Insomnia (*n* = 813)	*p*
Age, years	40.4 ± 10.3	39.9 ± 10.5	41.4 ± 10.0	0.001
Male gender, %	15.0	14.6	15.7	0.456
Smoking habit, %	34.6	37.2	29.5	<0.001
Months of work, median [IQR]	168 [72–300]	144 [72–300]	180 [84–312]	<0.001
Night shifts, %	99.7	99.7	99.6	0.642
Months spent in night shifts, median [IQR]	144 [60–264]	132 [60–252]	156 [60–276]	0.007
Night shifts per month, number	6.3 ± 1.4	6.3 ± 1.4	6.2 ± 1.4	0.163
Time to reach workplace, minutes	45 [45–65]	45 [44–65]	45 [45–65]	0.256
Rest time, minutes	180 [4–240]	180 [4–240]	180 [4–240]	0.821
Rest period after night shift, %	50.8	49.3	53.5	0.055
Rest in the afternoon, minutes	30 [0–120]	30 [0–120]	60 [0–120]	0.016
Number of coffees, mean	2.5 ± 1.5	2.5 ± 1.5	2.5 ± 1.4	0.698
Consumption of coffee during work, %	71.8	72.6	70.2	0.232
Number of coffees during night shift, mean	1.4 ± 1.1	1.4 ± 1.1	1.2 ± 1.5	0.178
Work in emergency department, %	38.4	37.6	39.9	0.250
History of insomnia previous to start working, %	25.7	29.4	18.7	<0.001
Referral to a Medical Specialist, %	9.0	10.2	6.8	0.006
Impairment (or new onset) of insomnia symptoms during COVID-19 period, %	75.2	82.5	61.4	<0.001
Insomnia symptoms impairment (or new onset) attributed to COVID-19 period, %	62.8	69.7	49.6	<0.001
Changing bedtime times during COVID-19 period, %	27.7	22.4	37.9	<0.001

**Table 2:** Multivariable logistic analyses on the correlates of insomnia in our study population. Model 1 encompasses all the variables with a *p* < 0.15 (for the association with insomnia) at univariate analysis. Model 2 was built after backward variable selection with elimination criterion of *p* ≤ 0.05.

Variables	Model 1		Model 2	
	Odds Ratio (95% CI)	*p*	Odds Ratio (95% CI)	*p*
Age (for 1 year)	0.99 (0.97–1.01)	0.464	0.98 (0.98–1.00)	0.014
Smokers (yes vs. no)	1.32 (1.09–1.60)	0.005	1.31 (1.09–1.59)	0.005
Months of work (for 1 month)	0.99 (0.99–1.01)	0.756	-	-
Months spent in night shifts (for 1 month)	1.00 (0.99–1.01)	0.993	-	-
Rest period after night shift (yes vs. no)	0.93 (0.77–1.11)	0.416	-	-
Rest in the afternoon (for 1 min)	0.99 (0.99–1.00)	0.059	0.99 (0.99–1.00)	0.050
History of insomnia previous to start working (yes vs. no)	1.70 (1.37–2.12)	<0.001	1.71 (1.37–2.11)	<0.001
Referral to a Medical Specialist (yes vs. no)	1.51 (1.08–2.11)	0.017	1.50 (1.07–2.11)	0.017
Impairment (or new onset) of insomnia symptoms during COVID-19 period (yes vs. no)	2.06 (1.61–2.64)	<0.001	2.07 (1.61–2.65)	<0.001
Insomnia symptoms impairment (or new onset) attributed to COVID-19 period (yes vs. no)	1.52 (1.21–1.91)	<0.001	1.51 (1.21–1.90)	<0.001
Changing bedtime times during COVID-19 period (yes vs. no)	0.53 (0.44–0.64)	<0.001	0.52 (0.43–0.64)	<0.001

## Data Availability

The data presented in this study are available on request from the corresponding author. The data are not publicly available because an electronic link to the data has not been created.
